# Supplementary material for: Polynomial Mendelian randomization reveals non-linear causal effects for obesity-related traits
Source: HGG Adv. 2022 Jun 22;3(3):100124. doi: 10.1016/j.xhgg.2022.100124 (PMC9272036; doi:10.1016/j.xhgg.2022.100124)
Supplement: Document S2. Article plus supplemental information [file mmc2.pdf]

HGG Advances

ARTICLE

Polynomial Mendelian randomization reveals non-linear causal effects for obesity-related traits

Jonathan Sulc,[1,2] Jennifer Sjaarda,[1,2] and Zoltán Kutalik[1,2,3,*]

Abstract

Causal inference is a critical step in improving our understanding of biological processes, and Mendelian randomization (MR) has emerged as one of the foremost methods to efficiently interrogate diverse hypotheses using large-scale, observational data from biobanks. Although many extensions have been developed to address the three core assumptions of MR-based causal inference (relevance, exclusion restriction, and exchangeability), most approaches implicitly assume that any putative causal effect is linear. Here, we propose PolyMR, an MR-based method that provides a polynomial approximation of an (arbitrary) causal function between an exposure and an outcome. We show that this method provides accurate inference of the shape and magnitude of causal functions with greater accuracy than existing methods. We applied this method to data from the UK Biobank, testing for effects between anthropometric traits and continuous health-related phenotypes, and found most of these (84%) to have causal effects that deviate significantly from linear. These deviations ranged from slight attenuation at the extremes of the exposure distribution, to large changes in the magnitude of the effect across the range of the exposure (e.g., a 1 kg/m^2 change in BMI having stronger effects on glucose levels if the initial BMI was higher), to non-monotonic causal relationships (e.g., the effects of BMI on cholesterol forming an inverted U shape). Finally, we show that the linearity assumption of the causal effect may lead to the misinterpretation of health risks at the individual level or heterogeneous effect estimates when using cohorts with differing average exposure levels.

Introduction

Identifying factors that cause disease or influence disease progression is integral to both furthering our understanding of disease pathophysiology and helping inform the development of novel treatments and interventions. Randomized controlled trials (RCTs) are the gold standard for demonstrating such causality between an exposure and outcome; however, they are expensive, time consuming, and often unethical or infeasible. Mendelian randomization (MR) has proven to be an extremely reliable, cost-effective, and feasible alternative to RCTs to assess causality using large-scale observational genetic data. MR takes advantage of the fact that genetic variants are both determined at birth and inherited randomly and independently of other risk factors of a disease, using genetic variants as instrumental variables (IVs) to infer causality between an exposure and an outcome. This random allocation of genetic variants minimizes the possibility of reverse causality and confounding. MR has not only identified thousands of novel, causal relationships between risk factors and diseases but has also provided strong evidence for a non-causal effect of many other exposure-outcome relationships.[1]

MR relies on three core assumptions: (1) relevance (i.e., IVs must be associated with the exposure), (2) exchangeability (i.e., IVs must not be associated with any confounder in the exposure-outcome relationship), and (3) exclusion restriction (i.e., IVs must not affect the outcome except through the exposure). While many extensions of MR have been developed to address potential violations of the three core assumptions, nearly all MR approaches implicitly assume that any putative causal effect is linear in nature. However, the association between an exposure and an outcome is, in fact, often non-linear. For instance, the observed relationship between BMI and all-cause mortality has been repeatedly shown to be U shaped in nature, where an increased mortality risk exists on either side of the 20–24.9 kg/m^2 interval.[2] Additionally, many asymptotic relationships have been observed in the context of public health, whereby an intervention or risk factors appear to be beneficial or increase risk initially, and, subsequently, the effect plateaus after a certain threshold.[3,4] Along these lines, weight loss may reduce low-density lipoprotein (LDL) levels for only lean individuals due to an inverted U-shape relationship.[5] Currently, it is unclear whether these observations are merely an artifact of confounding and/or reverse causation or whether there is truly a causal relationship, non-linear in nature, underlying these observations.

While identifying causal relationships is of high research importance, of equal importance is a comprehensive understanding of the underlying mechanisms and dynamics. A biased and naive characterization of these causal relationships can lead to a misinformed understanding of disease mechanisms and ultimately misguided treatments and public-health recommendations and interventions.

[1]University Center for Primary Care and Public Health, University of Lausanne, Lausanne, Switzerland; [2]Swiss Institute of Bioinformatics, Lausanne, Switzerland; [3]Department of Computational Biology, University of Lausanne, Lausanne, Switzerland
*Correspondence: zoltan.kutalik@unil.ch
https://doi.org/10.1016/j.xhgg.2022.100124.
© 2022 The Author(s). This is an open access article under the CC BY license (http://creativecommons.org/licenses/by/4.0/).

To date, few approaches have been developed to investigate nonlinear causal relationships within an MR framework. Most approaches are semiparametric in nature and involve stratifying individuals based on the exposure distribution[6] or do not consider potential non-linear confounder effects.[7] The extra flexibility regarding the shape of the causal function offered by these semi-parametric methods comes at a cost of increased variance of the resulting estimator. To address these gaps, we developed PolyMR, an MR-based approach to assess non-linear causal relationships in a fully parametric fashion.

Materials and methods

Let X and Y denote two random variables representing complex traits. We intend to use MR to estimate a non-linear causal effect of X on Y. The genotype data of the SNPs to be used as IVs are denoted by G. To simplify notation, we assume that $E(X) = E(Y) = E(G) = 0$ and $Var(X) = Var(Y) = Var(G) = 1$. The effect sizes of the instruments on X are denoted by $\boldsymbol{\beta}$. Let us assume the following model:

$$X = G \cdot \beta + \epsilon_x$$
$$Y = f_\alpha(X) + \epsilon_y,$$

where the parametric function $f_\alpha(\cdot)$ determines the shape of the causal relationship between X and Y, and ϵ_x and ϵ_y are zero-mean errors. For simplicity, we assume that $f_\alpha(\cdot)$ is a polynomial—even if it is not, it can be approximated by one with arbitrary precision over the range of the majority of values X can take. For example, if we intend to test a quadratic causal relationship, $f_\alpha(x) = \alpha_0 + \alpha_1 \cdot x + \alpha_2 \cdot x^2$. Thus, the model can be rewritten as

$$X = G \cdot \beta + \epsilon_x$$
$$Y = \sum_{j=0}^{k} \alpha_j \cdot X^j + \epsilon_y.$$

The above equation can be expanded to

$$X = G \cdot \beta + \epsilon_x$$
$$Y = \sum_{j=0}^{k} \alpha_j \left(\sum_{s=0}^{j} \binom{j}{s} (G\beta)^s \cdot \epsilon_x^{j-s} \right) + \epsilon_y.$$

We rely on the INSIDE assumption,[8] which ensures that $cov(G\beta, \epsilon_y) = 0$. The error terms ϵ_x and ϵ_y can nevertheless be correlated because of a potential causal effect of Y on X (reverse causation) and/or due to confounders. Let us split ϵ_y into ϵ_x-dependent and -independent parts.

$$\epsilon_y = \left(\epsilon_y \middle| \epsilon_x, \epsilon_x^2, \ldots, \epsilon_x^l\right) + \tau_y = \sum_{j=0}^{l} r_j \cdot \epsilon_x^j + \tau_y$$

Since $cov(G\beta, \epsilon_x) = cov(G\boldsymbol{\beta}, \epsilon_y) = 0$, the residual noise τ_y is independent of both $G\beta$ and ϵ_x. As a consequence, $cov(X, \tau_y) = cov(X - G\beta, \tau_y) = 0$. This allows us to rewrite the main model equations:

$$X = G \cdot \beta + \epsilon_x$$
$$Y = \sum_{j=0}^{k} \alpha_j X^j + \sum_{j=0}^{l} r_j \epsilon_x^j + \tau_y = \sum_{j=0}^{k} \alpha_j X^j + \sum_{j=0}^{l} r_j (X - G\beta)^j + \tau_y.$$

The advantage of this equation system is that the error terms (ϵ_x and τ_y) are uncorrelated and independent of the respective explanatory variables.

Let the realizations of the random variables X, Y, G be denoted by $\boldsymbol{x}$, $\boldsymbol{y}$, $\boldsymbol{G}$, observed in a sample of size n. The parameters $\{\boldsymbol{\beta}, \boldsymbol{\alpha}, \boldsymbol{r}\}$ can be estimated by computing two ordinary least squares estimates, first estimating $\boldsymbol{\beta}$ using the first equation and substituting this into the second equation:

$$\widehat{\boldsymbol{\beta}} = (\boldsymbol{G}'\boldsymbol{G})^{-1} \cdot \boldsymbol{G}'\boldsymbol{x} \quad \text{(Equation 1)}$$

$$\begin{pmatrix} \widehat{\boldsymbol{\alpha}} \\ \widehat{\boldsymbol{r}} \end{pmatrix} = (M_0{}'M_0)^{-1} \cdot M_0{}'\boldsymbol{y} \quad \text{where} \quad M_0 : = \left[1, \boldsymbol{x}, \ldots, \boldsymbol{x}^k, (\boldsymbol{x} - \boldsymbol{G}\widehat{\boldsymbol{\beta}}), \ldots, (\boldsymbol{x} - \boldsymbol{G}\widehat{\boldsymbol{\beta}})^l\right] \quad \text{(Equation 2)}$$

The special case of this approach when $k = l = 1$ is equivalent to the standard control function approach. For higher orders, the terms k and l are not required to be equal, as k represents the powers of the $f_\alpha(\cdot)$ function describing the causal relationship between X and Y, whereas l represents the order of the function describing the effects of confounding and/or reverse causation, i.e. residual correction.

Implementation

We implemented this method in R. For the polynomial approximation of $f_\alpha(\cdot)$ function of the causal relationship, we included terms up to the 10th power for the exposure ($k = 10$) to allow sufficient flexibility for the shape of the causal function and included residual-correction terms up to the same order ($l = 10$). We then applied a backward model selection approach, iteratively eliminating exposure coefficients (α_j) that were not significant at a Bonferroni-corrected level ($0.05/k = 0.005$) by setting them to zero. Residual-correction terms (r_j) were retained up to the order of the highest remaining exposure coefficient. This ensures that any contribution of confounders to the high-order polynomial terms of the exposure-outcome relationship are not erroneously attributed to a causal effect. Once all remaining exposure coefficients were significant, the non-linearity p value was obtained with a likelihood ratio test (LRT), comparing the full model with that including only the linear effect but retaining all remaining residual correction terms. The causally explained variance was determined as the difference in explained variance (r^2) between the full model and that excluding all $\alpha_j \cdot \boldsymbol{x}_j$ terms (i.e., accounting only for potential confounding).

The polynomial function was the result of a multivariable regression, which also provided us with the variance-covariance matrix of the coefficient estimates. From these, we can generate causal polynomial functions whose coefficients are drawn from the established multivariable distribution to obtain the 95% confidence hull.

In order to avoid invalid IVs acting through reverse causation, we filtered out IVs where the standardized effect estimate was larger (in absolute value) in the outcome than in the exposure.

For comparison, LACE[6] was also implemented, and polynomial approximation was obtained in an analogous fashion. The piecewise linear LACE approach was not tested here but is considered in the discussion. We examined the limitations of the standard (1st order) control function approach by running PolyMR with $l = 1$, hereafter referred to as PolyMR-L1, in specific settings. We chose not to compare with results from CATE[7] as this method enables the estimation of differences in causal effects based on

exposure (i.e., slopes) but does not provide an overall function and makes comparison difficult. Furthermore, the implementation available at the time of this writing was comparatively slow and did not scale well when tested on biobank-size data: the estimated run time for a single simulation using our parameters, repeated at ~100 exposure levels to model function shape, was several days for fewer than 10 IVs.

Simulations

We simulated data according to the following model:

$$X = G \cdot \beta + q_x \cdot U + \epsilon_x$$
$$Y = \alpha_1 X + \alpha_2 \cdot X^2 + q_y \cdot U + q_{y2} \cdot U^2 + \epsilon_y,$$

where U is a confounder drawn from a standard normal distribution. Columns of G were drawn from a binomial distribution with minor allele frequencies following a beta distribution with shape parameters equal to 1 and 3, and then we normalized each column to have zero mean and unit variance. The genetic effects β_i were drawn from normal distributions based on the minor allele frequencies, specifically $\beta_i \sim \mathcal{N}(0, (p_i * (1 - p_i))^{-0.25})$, where p_i is the minor allele frequency of SNP i, and scaled such that the total explained variance matches the predefined heritability, i.e., $\sum \beta_i^2 = h^2$. These effect sizes are realistic and are according to the baseline LDAK heritability model (without functional categories) with a selection strength of -0.25.[9,10] For the basic settings, we included moderate confounding ($q_x = 0.2$, $q_y = 0.5$, $q_{y2} = 0$) and a quadratic causal function ($f_\alpha(X) = 0.1X + 0.05X^2$). The heritability h^2 was set to 0.5, explained by $m = 100$ causal SNPs, and the sample size was set to 100,000 individuals. Causal SNPs were filtered for genome-wide significance of their marginal effects in the simulated data prior to their use as IVs. Note that due to lack of statistical power, most of the causal SNPs do not reach genome-wide significance and hence are not used as instruments. Variations on these settings were tested, as shown in Table 1. Each combination of parameters was used to generate 1,000 sets of data, to which we applied both PolyMR and LACE. We compared these with the performance of PolyMR-L1 in the base settings and in the presence of weak quadratic confounding ($q_x \times q_{y2} = 0.04$), as well as in the absence of quadratic causal effect but with quadratic confounding ($q_x \times q_{y2} = 0.1$), creating a similar observed association between traits as in the base settings.

The theoretical 95% confidence hulls were compared with the empirical distribution of estimated models. At each percentile of the exposure distribution, the size of the predicted 95% confidence intervals (CIs) was compared with the empirical one.

Application to UK Biobank data

The UK Biobank is a prospective cohort of over 500,000 participants recruited in 2006–2010 and aged 40–69.[11] We tested for non-linear causal effects of anthropometric traits (body mass index [BMI], weight, body-fat percentage [BFP], and waist-to-hip ratio [WHR]) on continuous health outcomes (pulse rate [PR], systolic blood pressure [SBP], diastolic blood pressure [DBP], glucose, low-density lipoprotein [LDL], high-density lipoprotein [HDL], and total cholesterol [TC] levels in blood), as well as the reverse. We also tested for effects of both BMI and age completed full-time education on life expectancy. To boost statistical power, we applied the idea of the kin-cohort design, where parental lifespan is used as a proxy for the participant's lifespan.[12] Since most participants in the UK Biobank are still alive, we scaled the mother's and father's age of death (separately) and used the mean of these standardized phenotypes as a proxy for the individual's life expectancy. For participants with one parent still alive, we used the other's age of death. Participants with both parents still alive were excluded for this particular analysis.

We selected 377,607 unrelated White British participants, and all phenotypes were corrected for age, age^2, sex, age × sex, and age^2 × sex as well as the top 10 genetic principal components. With the exception of WHR, IVs were selected using the TwoSampleMR R package[13] (v.0.5.5) with default settings ($p < 5 \times 10^{-8}$, $r^2 < 10^{-3}$, $d > 10^4$ kb) from the genome-wide association study (GWAS) in the ieugwasr R package[14] (v. 0.1.5) with the largest number of instruments overlapping our dataset. For WHR, we used a previously performed GWAS on the aforementioned sample from the UK Biobank, adjusting for covariates as above.[15]

For the purpose of comparison, we also used inverse-variance weighted MR and MR Egger on each of these exposure-outcome pairs. These were performed with the same IVs and (in-sample) association statistics using the TwoSampleMR R package[13] (v.0.5.5). We also compared the results of standard PolyMR with those PolyMR-L1 to determine whether accounting for higher-order confounding is necessary in real data applications.

Results

Simulations

We simulated a variety of settings, including many combinations of heritability and polygenicity in the exposure, sample size, and shape of the causal function $f_\alpha(\cdot)$ and confounding. Where the true underlying function was polynomial, our approach correctly captured its shape (Figure 1), although a slight bias from confounding was introduced in certain settings with high polygenicity (>1,000 causal SNPs) or strong confounding (where linear confounding alone would lead to a correlation of 0.4 between the exposure and the outcome, i.e., $q_x = 0.5$, $q_y = 0.8$) (Figure 2). The distribution of this bias was affected by the shape of the confounding, i.e., in situations with quadratic confounding, the bias was quadratic with respect to exposure. In all simulation settings, this bias was orders of magnitude smaller than both the causal effect and the confounding (e.g., Figure 1A). Although this bias was minimal with the standard PolyMR settings ($l = k$), quadratic confounding produced significant bias when higher orders of the control function term ($\boldsymbol{x} - \boldsymbol{G}\widehat{\boldsymbol{\beta}}$) were ignored (i.e., $l = 1$ in PolyMR-L1; Figure S1), which is the standard approach for control function use.

In the case of non-polynomial functions, PolyMR nevertheless provided reasonable estimates of the true shape of the causal function (Figure 1B). The bias introduced in these cases (Figures 1B and 2B) is consistent with expectations of polynomial approximation with limited power and is dependent on the shape of the non-polynomial function.

LACE also produced some bias in estimating the causal function. The magnitude of the bias introduced by either method was dependent on the settings used, with the bias being generally larger when the per-SNP heritability

Table 1. Causal functions ($f_\alpha(\cdot)$) and setting parameter combinations simulated for PolyMR

Causal functions	$f_\alpha(X)$
Base settings[a]	$0.1 \cdot X + 0.05 \cdot X^2$
Null	0
Linear effect	$0.1 \cdot X + 0 \cdot X^2$
Stronger effect	$0.3 \cdot X + 0.1 \cdot X^2$
Weak quadratic effect	$0.1 \cdot X + 0.01 \cdot X^2$
Cubic effect	$0.1 \cdot X + 0.05 \cdot X^2 + 0.05 \cdot X^3$
Fourth-order effect	$0.1 \cdot X + 0.05 \cdot X^2 + 0.05 \cdot X^4$
Third- and fourth-order effects	$0.1 \cdot X + 0.05 \cdot X^2 + 0.03 \cdot X^3 + 0.01 \cdot X^4$
Exponential effect	$0.1 \cdot e^X$
Square root effect	$0.1 \cdot sgn(X) \cdot \sqrt{\lvert X \rvert}$
Sigmoid effect (1)	$0.1 \cdot \frac{1}{1+e^{-X}}$
Sigmoid effect (2)	$0.1 \cdot \frac{1}{1+e^{-2 \cdot X}}$
Sigmoid effect (3)	$0.1 \cdot \frac{1}{1+e^{-3 \cdot X}}$
Other settings	
Strong confounding	$q_x = 0.5, q_y = 0.8$
Negative confounding	$q_y = -0.5$
Quadratic confounding[a]	$q_{y2} \cdot U^2$ term added to Y, where $q_{y2} \in \{0.1, 0.2\}$
[a]linear effect Quadratic confounding,	$0.2 \cdot U^2$ term added to Y, $f_\alpha(X) = 0.1 \cdot X$
Heritability and polygenicity	$h^2 \in \{0.2, 0.3, 0.5, 0.8\}$, $m \in \{20, 100, 1K, 5K, 10K\}$

[a]Settings where PolyMR-L1 was also applied for comparison.

was lower (i.e., settings with higher polygenicity). Another key contributor to increased bias was the non-polynomial nature of the underlying causal function. In some cases, the coefficient selection procedure led to greater bias from LACE due to lack of statistical power and fewer coefficients being selected. In all settings tested, PolyMR still provided lower bias (Figure S2) and root-mean-square errors (RMSEs) than LACE (Figure 3), partly driven by greater statistical power and smaller SEs.

To ensure that the variance estimated from the variance-covariance matrix of the model was correctly calibrated, we assessed the coverage of the 95% CIs. We did so by comparing the predicted 95% CIs of the curves with those derived empirically from repeated simulations. We found that in the case of most polynomial functions, the CIs were properly calibrated, with the theoretical and empirical CIs being almost equal across most of the exposure distribution. Note that under some simulation settings (e.g., weak quadratic effects, $\alpha_2 = 0.01$), allowing the polynomial degree to vary led to increased empirical variance (see Figure S3A). However, if we consider only those simulations where the second order was correctly inferred (920 simulation results out of 1,000), the empirical CIs were close to those predicted by the method (Figure S3B).

UK Biobank

Given its favorable performance throughout all simulation settings, we applied the PolyMR method to data from the UK Biobank. We set out to estimate the causal effects of four anthropometric traits (BMI, weight, BFP, and WHR) on each of seven continuous traits commonly used as health biomarkers (SBP, DBP, PR, and the levels of glucose, HDL, LDL, and TC in the blood). We also tested for reverse causal effects for these trait pairs as well as any effects of BMI or education on life expectancy.

The effects of the anthropometric traits were qualitatively similar to one another and significant against all tested outcomes, with significant non-linearity in most cases (Figures S4–S31). Those of BFP and WHR tended to be more similar to one another, monotonically increasing DBP, SBP, and PR with linear to slightly non-linear effects. BMI also increased these traits overall, though the effects of BMI on DBP and SBP plateaued at around 2 SD above the population mean (~36.9 kg/m^2), and the causal function for BMI on PR showed a positive slope for values between approximately 1 SD below to 2 SDs above the population mean (~22.7–36.9 kg/m^2), with negative slopes beyond these. The effects of weight were weaker but qualitatively similar to those of BMI. Glucose was increased by all

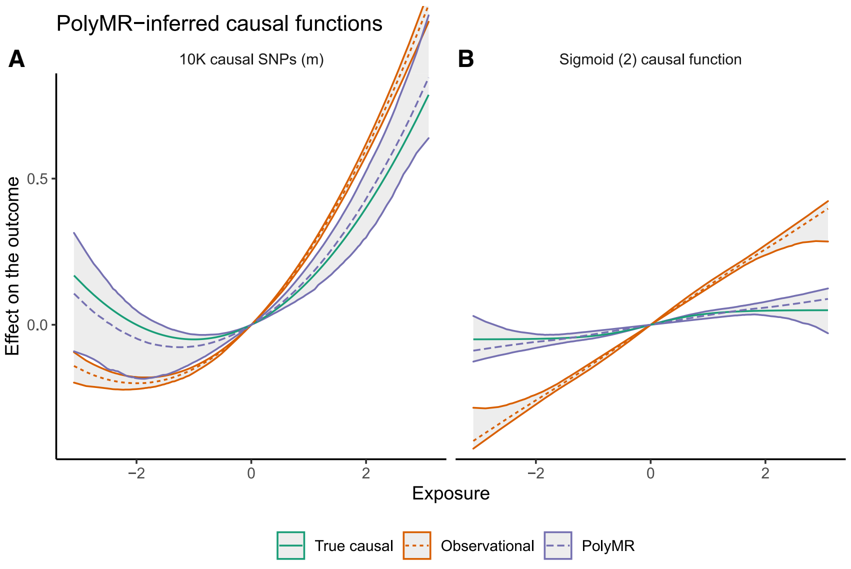

Figure 1. PolyMR is able to recover the shape of the causal function
The true causal function is shown in green (solid line). The observed association model is shown in orange (short dashed) while that obtained using PolyMR is shown in purple (long dashed). The hulls around the model curves show the 95% coverage hull across 1,000 simulations. The hulls around the observational effects represent the 95% confidence interval estimated from the conditional distribution of the outcome. The y axis shows the expected association with/effect of the exposure on the outcome, relative to the outcome level at the mean population exposure. (A) A setting representing high polygenicity (10,000 causal SNPs accounting for a heritability of 0.3; see Table 1 for details). (B) Results for a sigmoid causal effect ($f_\alpha(X) = 0.1 \cdot \frac{1}{1+e^{-2 \cdot X}}$ scenario.

of these, though the effects of a change in exposure were negligible below −1 SD for all traits and intensified at higher values. For example, the estimated slope of the standardized effect of BMI was 0.17 at the population mean but increased to 0.31 at +2 SD. The strongest non-linearity in the effects of anthropometric traits was found for TC, mainly driven by the LDL fraction (Figure 4A), where the causal function took a strong inverted U shape. In contrast to this, their effects on HDL were all monotonic decreasing.

The consequences of PR were limited to weak linear effects on BFP and WHR. TC linearly decreased BMI, weight, and BFP, with no detectable effect on WHR. SBP and DBP both had inverted U-shaped causal functions for their effects on all anthropometric traits ($p < 1.6 \times 10^{-47}$), with the effects on WHR being slightly weaker. Glucose levels had nearly no effect on most traits across most of the distribution but drove strong reductions at higher values, with the exception of WHR, which was in fact slightly increased by glucose levels up to ~ 3 SD before being decreased at higher levels. HDL had a slight U-shaped effect on these traits, with a stronger increase for high values of the exposure on BFP and no increase in WHR.

Although the observational association of SBP/DBP and the anthropometric traits was mostly monotonic increasing, the estimated causal function on these had an inverted U shape (Figure 4B), with slightly weaker effects on WHR. The causal effects of PR on anthropometric traits show a slight positive slope close to the population median, but the directionality switches at either extreme of the distribution. LDL cholesterol decreased the outcomes near monotonically, though the effect close to the population median was weak to null (Figure 4C). The impact of glucose was slightly different across the anthropometric traits. Both BMI and weight were overall negatively affected by glucose levels (with weaker effects around zero). BFP was also decreased, although the effect was much weaker. WHR, however, was slightly increased by glucose levels up to ~ 3 SD before being decreased at higher levels. Note that the effect close to the population mean is likely driven by a decrease in hip circumference rather than an increase in the waist's, similar to what we have shown previously for the effects of diabetes risk and triglyceride levels on WHR-related metrics.[15]

The effects of BMI and education on life expectancy are directionally as expected, but we found no evidence of non-linearity. The BMI-life expectancy (causal) relationship was decreasing, though the intensity of the effect was greater than the observed association (Figure 4D). As expected, higher education increased life expectancy, but we found no evidence of non-linearity in the effect (Figure S32).

The exclusion of higher-order control function terms in PolyMR-L1 produced somewhat different inferred causal functions, with generally stronger non-linear components, resulting in inferred causal functions that were closer to the observed associations (Figures S33–S39). These linear control functions are used in competing methods,[7,16] which are outperformed by PolyMR in such settings.

Discussion

In this report, we present PolyMR, an MR-based approach for the inference of non-linear causal effects. Through a variety of simulations, we showed that it is robust to many forms of confounding and is well powered to detect even weak quadratic effects in biobank-size cohorts. Finally, by applying our method to the UK Biobank, we showed that causal effects across many anthropometric traits indeed include strong non-linear components.

Despite statistically significant non-linearity for the causal effects of many exposures on outcomes, some of these were monotonic or even near linear around the population median. In these cases, the causal effect estimates from traditional, linear, MR methods will likely still be useful, though non-linearity in the tails of the distribution

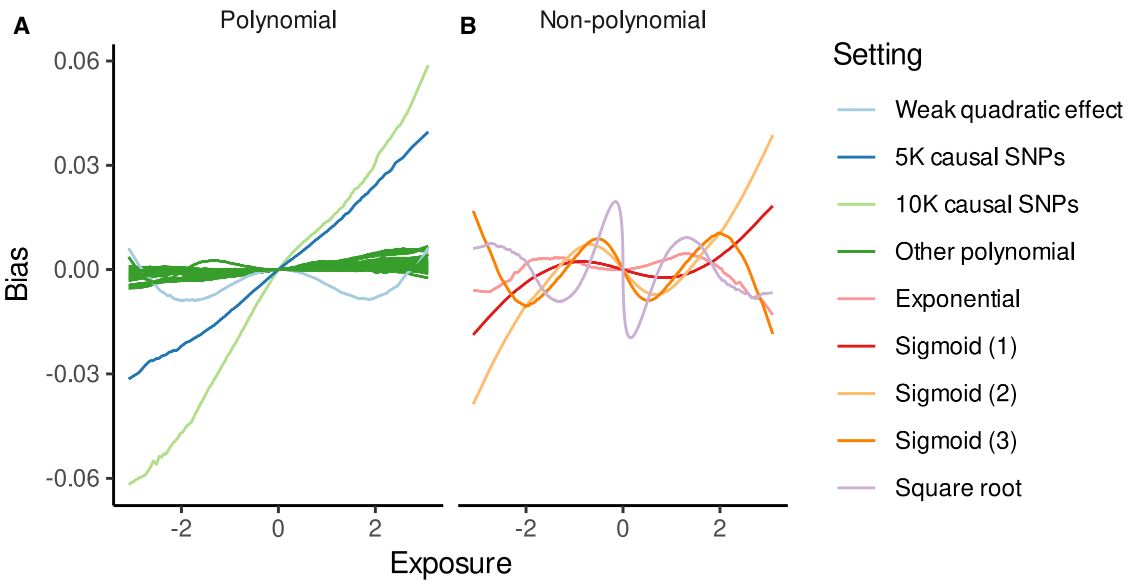

Figure 2. Bias in the causal effect estimation as a function of exposure across settings
(A) In settings with a polynomial causal function $f_\alpha(\cdot)$, slight bias from non-genetic confounding was induced under certain combinations of high polygenicity, high heritability, or strong confounding. (B) The bias found in non-polynomial settings was expected due to the polynomial approximation approach.

may introduce varying amounts of bias for certain exposure levels. Even where the overall causal effect is monotonic and could therefore be described as "positive" or "negative," knowing the shape of the curve can provide insight into which strata of the population may benefit most from public-health interventions. For example, weight loss for individuals with average BMI is far more beneficial in in terms of lowering SBP than it is for obese individuals. The bias introduced by the assumption of linearity increases further with non-monotonic effects, such as that of SBP on BMI, and the effect estimates vary greatly based on the method used, but without proper consideration of the non-linear components, it will not be particularly meaningful. Although in certain contexts, a linear approximation of the monotonic effects can be useful, they will introduce bias. Specifically, different populations with different mean values of exposure will yield different linear estimates even in cases where there is true causality and all IVs are valid.

There are a number of possible explanations for the non-linear causal relationships we identified, particularly in the case of the inverted U-shaped curves, where both the exposure and outcome are presumed negative markers of health (for example, BMI and cholesterol). The shape of these effects could arise from several mechanisms such as negative feedback loops. The most obvious, but intangible, candidates are biological feedback mechanisms. A more concrete possibility is a lifestyle change in response to elevated risk, by either doctor recommendation, medication, or personal or social pressures. Either off- or on-target effects of these changes could play a role on the inverted U-shaped curves we identified. These latter effects, however, are expected to be weak and explain only a small part of these phenomena.
Another explanation may be interaction effects between an exposure-associated environmental variable and the exposure itself influencing the outcome.

We identified several benefits of our method compared with existing tools such as LACE and CATE. PolyMR not only demonstrated greater accuracy than LACE, but it does not require arbitrary choices regarding bin numbers and spacing like LACE does. The other competing method, CATE, assumes that any sources of confounding are linear and will introduce bias/false positives in the presence of sources of non-linear confounding, whereas polyMR allows for non-linear sources of confounding. Of note, while CATE can estimate the change in outcome based on the difference in exposure, it does not explicitly estimate the shape of the causal function. More general methods, based on kernel ridge regression,[17] lack software implementation and, due to their computational complexity, may not be applicable to sample sizes >100,000.

This work has certain limitations that should be taken into account. First, our method requires individual-level data. While using classical summary statistics, non-linear causal effects are undetectable; however, one could envision approximating the polyMR method by using not only $G-X$ and $G-Y$ association summary statistics but higher-order ($G^i - Y$, $G^i - X^j$, $X^i - Y$, and $G^i \cdot X^j - Y$ with $i,j = 0,1,2,\ldots,k$) associations. Although such an approximation would require additional summary statistics, those are not generally available for any trait and hence would not facilitate its use in practice. Secondly, the estimates provided for the extremes of the distribution are less reliable, and removing outliers may improve the reliability of estimates across the entire distribution. Third, a small amount of bias is introduced due to the fact that we use

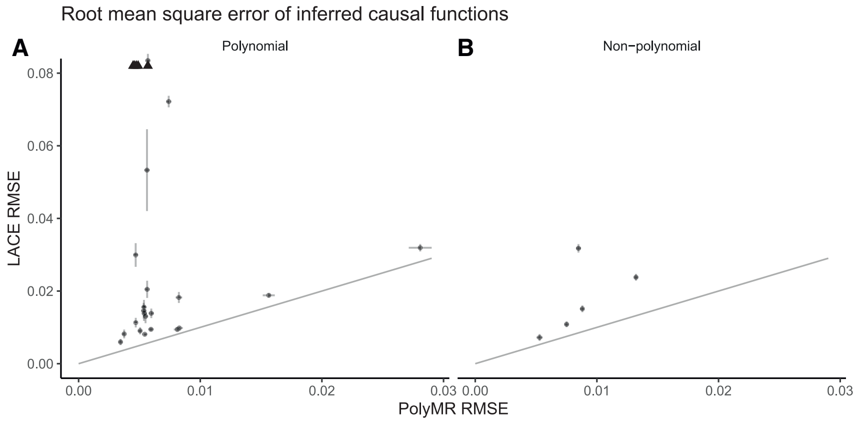

Figure 3. PolyMR provided greater accuracy in the estimation of causal functions
The root-mean-square errors (RMSEs) are shown for both PolyMR and LACE. Each point is the mean RMSE for a given setting, with the error bars showing the 95% confidence interval of the mean. (A) Settings for polynomial causal functions. (B) Settings for non-polynomial causal functions. Arrows in (A) indicate RMSEs that exceed the bounds of the plot.

$G \cdot \widehat{\beta}$ instead of $G \cdot \beta$ in our model fitting (Equation 2). To mitigate this, maximum likelihood estimation (MLE) could also be used to take into account the error in SNP-exposure associations. Fourth, our approach still suffers from the weaknesses of classical MR methods, such as Winner's curse and invalidity of the instruments. Winner's curse could be addressed by splitting the sample and using one subset to select instruments and estimate their effects, while the other subset would be used for the rest of the polyMR algorithm. Such a solution would reduce bias, along with decreased power. Fifth, the true causal function may well be non-polynomial, but still the polynomial approximation for the bulk of the exposure range (e.g. [−2SD,2SD]) can provide a firm idea about the shape of the curve, even if the actual coefficients and the behavior of the curve beyond the exposure extremes are meaningless. Sixth, our simulations have not explored violations of the InSIDE assumption, which may have more drastic consequences for non-linear MR. Seventh, the backward selection process to settle on the optimal polynomial may suffer from post-selection inference, which could alter the coverage of the 95% CIs. However, in our simulations, this did not seem to noticeably influence the coverage, and backward selection can be disabled in the implemented function if preferred. Finally, although this method could be generalized to binary outcomes, it has limited utility. The shape of such non-linear relationships would largely depend on the link function used for the generalized linear models, while showing deviations from a linear relationship for continuous outcomes reveals not only a quantitatively better fitting model but also a qualitatively different one. However, for binary outcomes, even if there is a non-linear term, the model class remains qualitatively similar (since any link function is already non-linear),

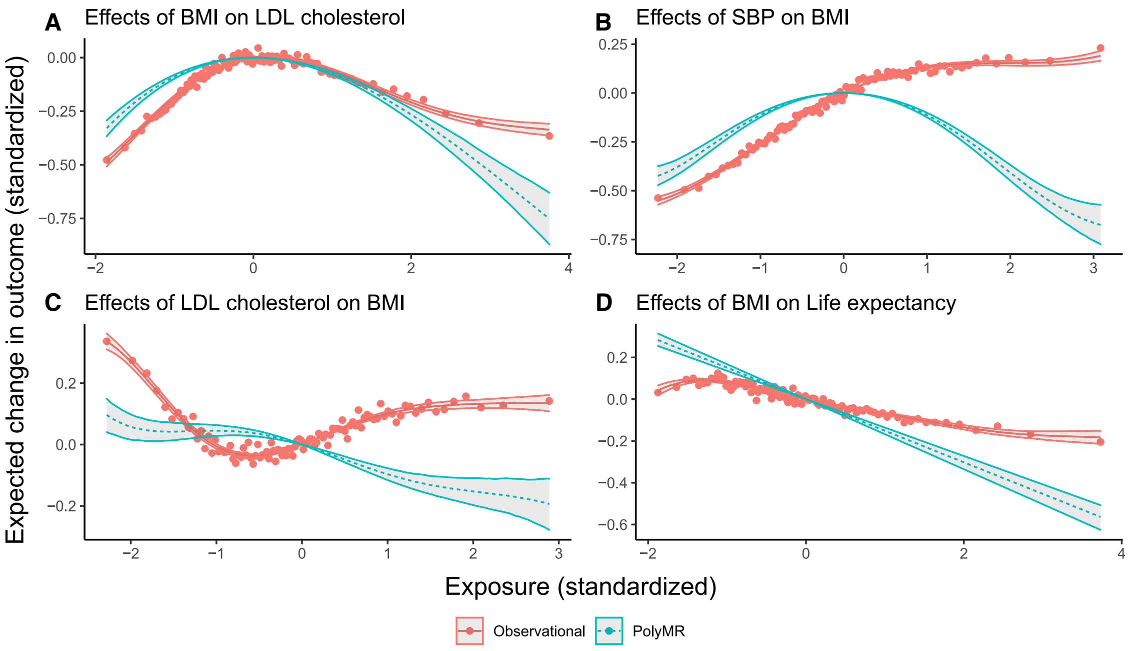

Figure 4. Most tested causal effects have strong non-linear components in the UK Biobank
The red points show the mean outcome plotted against the median exposure for each of 100 bins, split by covariate-adjusted exposure level. The red curve (solid) is the multivariable regression model, whereas the teal one (dashed) corresponds to the estimated causal function obtained using PolyMR. The hulls around both curves correspond to the 95% confidence interval. (A–D) Four trait pairs: (A) BMI on LDL cholesterol, (B) SBP on BMI, (C) LDL cholesterol on BMI, and (D) BMI on life expectancy.

and it only indicates that the causal relationship could be better described with a different kind of link function. The only exception to this is when the causal function is non-monotonic, since link functions are strictly monotonically increasing. Therefore, the method is best suited to continuous traits but may also reveal interesting insights for non-monotonic causal functions for binary outcomes.

In summary, we have developed an MR approach for the estimation of non-linear exposure-outcome causal effects. We have shown the utility of this approach when applied to cardiovascular and anthropometric traits in the UK Biobank, where we identified numerous relationships that show significant deviations from linearity. Indeed, non-linear effects are pervasive in biology and should be considered appropriately when developing public-health policies. Future studies should investigate the impact of non-linear causal effects on the complete human phenome to determine the prevalence of non-linear causal relationships among other conditions and biological pathways. PolyMR allows for a more nuanced picture of causal mechanisms beyond mere identification of causal factors for disease. Better understanding of such complex, non-linear relationships will help make better-informed health interventions.

Data and code availability

There are restrictions to the availability of the UK Biobank data due to ethical/privacy considerations, but they are available through an application procedure (see web resources). The published article includes all resulting data generated during this study. The source code generated during this study is available at https://github.com/JonSulc/PolyMR.

Supplemental information

Supplemental information can be found online at https://doi.org/10.1016/j.xhgg.2022.100124.

Acknowledgments

This research has been conducted using the UK Biobank resource (#16389), which has been approved by the National Research Ethics Service Committee. The computations have been carried out on the HPC server of the Lausanne University Hospital. Z.K. was funded by the Swiss National Science Foundation (31003A-143914 and 310030-189147).

Author contributions

Z.K. and J. Sulc conceived the method. J. Sulc performed the simulation studies, analysis of real data, and wrote the initial draft of the paper with contributions from J. Sjaarda and Z.K. All authors have read and approved the manuscript.

Declaration of interests

The authors declare no competing interests.

Received: March 24, 2022
Accepted: June 13, 2022

Web resources

ieugwasr, https://github.com/MRCIEU/ieugwasr
Source code, https://github.com/JonSulc/PolyMR
UK Biobank, http://www.ukbiobank.ac.uk/using-the-resource/

References

1. Vanderweele, Tyler J., T Tchetgen, E.J., Cornelis, M., and Kraft, P. (2014). Methodological challenges in Mendelian randomization. Epidemiology *25*, 427–435.
2. Berrington de Gonzalez, A., Hartge, P., James R, Cerhan, Alan, J. Flint, Hannan, L., MacInnis, R.J., Moore, S.C., T, G.S., Anton-Culver, H., Laura Beane, Freeman, et al. (2010). Body-mass index and mortality among 1.46 million white adults. N. Engl. J. Med. *363*, 2211–2219.
3. Malik, R., Georgakis, M.K., Vujkovic, M., Damrauer, S.M., Elliott, P., Karhunen, V., Giontella, A., Fava, C., Hellwege, J.N., Shuey, M.M., et al. (2021). Relationship between blood pressure and incident cardiovascular disease: linear and nonlinear mendelian randomization analyses. Hypertension *77*, 2004–2013.
4. Stavrova, O., and Ren, D. (2021). Is more always better? Examining the nonlinear association of social contact frequency with physical health and longevity. Soc. Psychol. Personal. Sci. *12*, 1058–1070.
5. Laclaustra, M., Lopez-Garcia, E., Civeira, F., Garcia-Esquinas, E., Graciani, A., Guallar-Castillon, P., Banegas, J.R., and Rodriguez-Artalejo, F. (2018). LDL cholesterol rises with BMI only in lean individuals: cross-sectional U.S. And Spanish representative data. Diabetes Care *41*, 2195–2201.
6. Staley, J.R., and Burgess, S. (2017). Semiparametric methods for estimation of a nonlinear exposure-outcome relationship using instrumental variables with application to Mendelian randomization. Genet. Epidemiol. *41*, 341–352.
7. S. Li and Z. Guo. Causal Inference for Nonlinear Outcome Models with Possibly Invalid Instrumental Variables, 2020.
8. Bowden, J., George, D. Smith, and Burgess, S. (2015). Mendelian randomization with invalid instruments: effect estimation and bias detection through Egger regression. Int. J. Epidemiol. *44*, 512–525.
9. Speed, D., Cai, N., Johnson, M.R., Nejentsev, S., and B, D.J. (2017). Reevaluation of SNP heritability in complex human traits. Nat. Genet. *49*, 986–992.
10. Speed, D., Holmes, J., and David, J. (2020). Balding. Evaluating and improving heritability models using summary statistics. Nat. Genet. *52*, 458–462.
11. Sudlow, C., Gallacher, J., Allen, N., Beral, V., Burton, P., Danesh, J., Paul, D., Elliott, P., Green, J., Landray, M., et al. (mar 2015). UK biobank: an open access resource for identifying the causes of a wide range of complex diseases of middle and old age. PLoS Med. *12*, e1001779.
12. Timmers, P.R.H.J., Mounier, N., Lall, K., Fischer, K., Zheng, N., Xiao, F., Bretherick, A.D., Clark, D.W., Shen, X., Esko, T., et al. (2019). Genomics of 1 million parent lifespans implicates novel pathways and common diseases and distinguishes survival chances. Elife *8*, e39856.

13. Hemani, G., Zheng, J., Elsworth, B., Wade, K.H., Haberland, V., Baird, D., Laurin, C., Burgess, S., Bowden, J., Ryan, L., et al. (2018). The MR-Base platform supports systematic causal inference across the human phenome. Elife *7*.
14. G. Hemani. MRCIEU/ieugwasr: R Interface to the IEU GWAS Database API.
15. Sulc, J., Anthony, S., Mounier, N., Auwerx, C., Marouli, E., Darrous, L., Draganski, B., Kilpeläinen, T.O., Joshi, P., Ruth, J., et al. (2021). Composite trait Mendelian randomization reveals distinct metabolic and lifestyle consequences of differences in body shape. Commun. Biol. *4*, 1–13.
16. Guo, Z., and Dylan, S. (2016). Small. Control function instrumental variable estimation of nonlinear causal effect models. J. Mach. Learn. Res. *17*, 3448–3482.
17. Singh, R., Sahani, M., and Gretton, A. (2019). Kernel Instrumental Variable Regression (Curran Associates Inc.).

HGGA, Volume *3*

Supplemental information

Polynomial Mendelian randomization reveals non-linear causal effects for obesity-related traits

Jonathan Sulc, Jennifer Sjaarda, and Zoltán Kutalik

Supplementary figures

November 26, 2021

Contents

1 Simulations **2**

2 Application in the UK Biobank **5**
2.1 Body fat percentage 5
2.2 Waist-to-hip ratio 12
2.3 BMI 19
2.4 Weight 26
2.5 Education and life expectancy 33

3 PolyMR-L1 **34**
3.1 BMI 34

1 Simulations

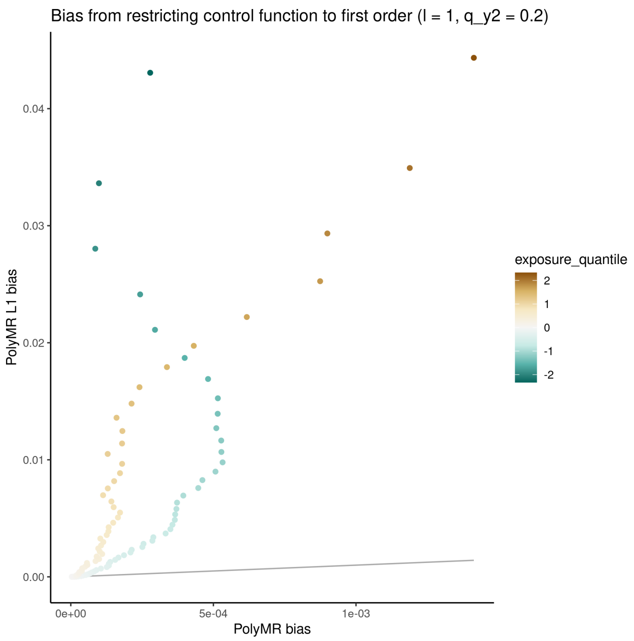

Supplementary figure 1: Not accounting for higher order confounding introduces bias. The (absolute) biases are shown for both PolyMR and PolyMR-L1 in the setting with quadratic confounding. Each point is the mean absolute bias across simulations for a given percentile of the exposure distribution.

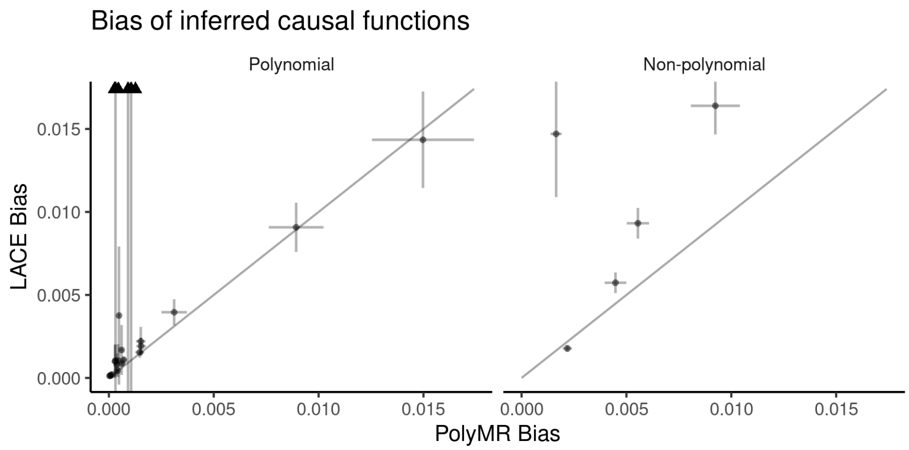

Supplementary figure 2: PolyMR generally yields less bias than LACE. The (absolute) biases are shown for both PolyMR and LACE. Each point is the mean absolute bias across simulations for a given setting with the error bars showing the 95% confidence interval of the mean. Settings were split into polynomial or non-polynomial causal functions. Arrows in the polynomial plot indicate mean biases exceeding the bounds of the plot.

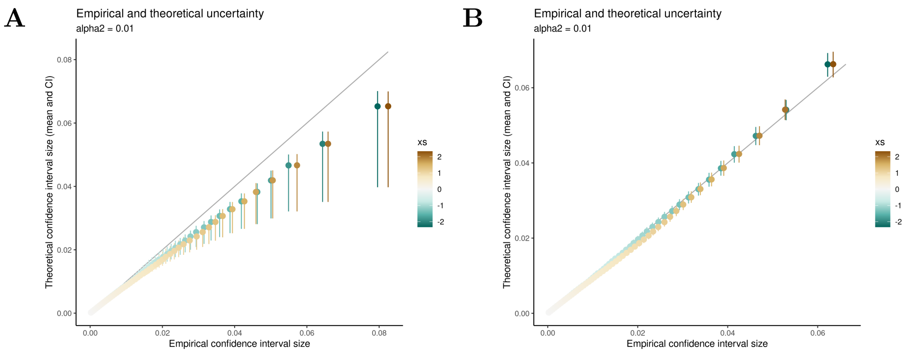

Supplementary figure 3: The confidence intervals (CIs) of PolyMR are correctly calibrated for the given model but may be slightly underestimated due to post-selection inference if the model is allowed to vary. The size of the theoretical 95% CI was calculated for each simulation at each percentile of the exposure, yielding a distribution of CI sizes. The empirical CIs come from the distribution of coefficient estimates across simulations. Both panels show the CIs for the weak quadratic effect setting, with either (A) all results or (B) only those results where the correct order of the causal function was determined (920 out of 1000 simulations).

2 Application in the UK Biobank

2.1 Body fat percentage

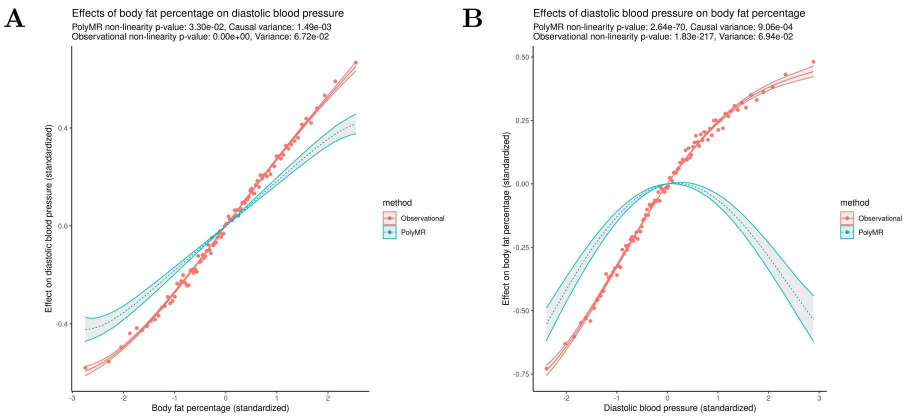

Supplementary figure 4: The observed (red, continuous) and causal (blue, dashed) association between body fat percentage and diastolic blood pressure (A) and the reverse (B). The observed association (red, continuous) was estimated using a similar polynomial approximation for the purpose of comparison, the points showing the mean outcome level at each percentile of the exposure. The hulls surrounding both functions show the 95% confidence range across the distribution.

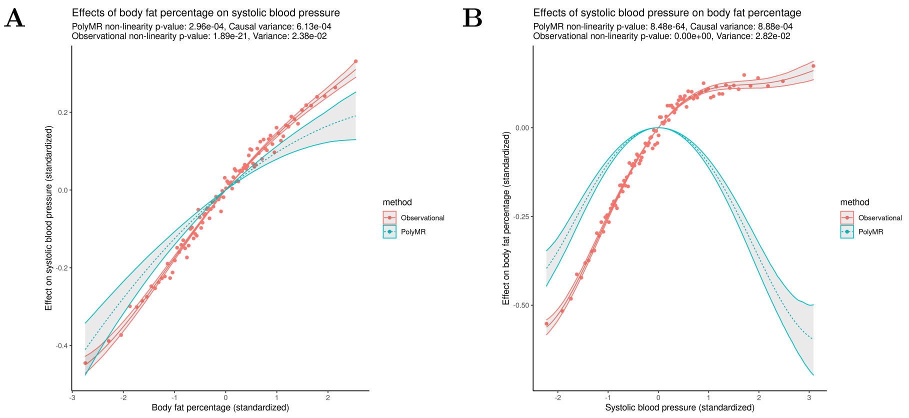

Supplementary figure 5: The observed (red, continuous) and causal (blue, dashed) association between body fat percentage and systolic blood pressure (A) and the reverse (B). The observed association (red, continuous) was estimated using a similar polynomial approximation for the purpose of comparison, the points showing the mean outcome level at each percentile of the exposure. The hulls surrounding both functions show the 95% confidence range across the distribution.

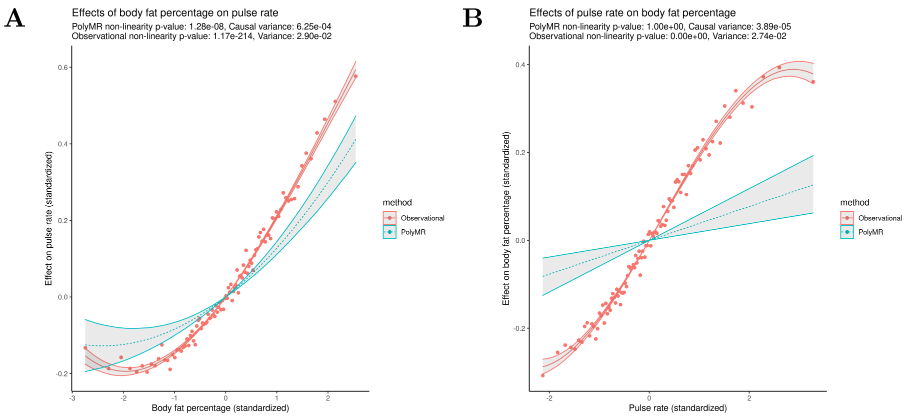

Supplementary figure 6: The observed (red, continuous) and causal (blue, dashed) association between body fat percentage and pulse rate (A) and the reverse (B). The observed association (red, continuous) was estimated using a similar polynomial approximation for the purpose of comparison, the points showing the mean outcome level at each percentile of the exposure. The hulls surrounding both functions show the 95% confidence range across the distribution.

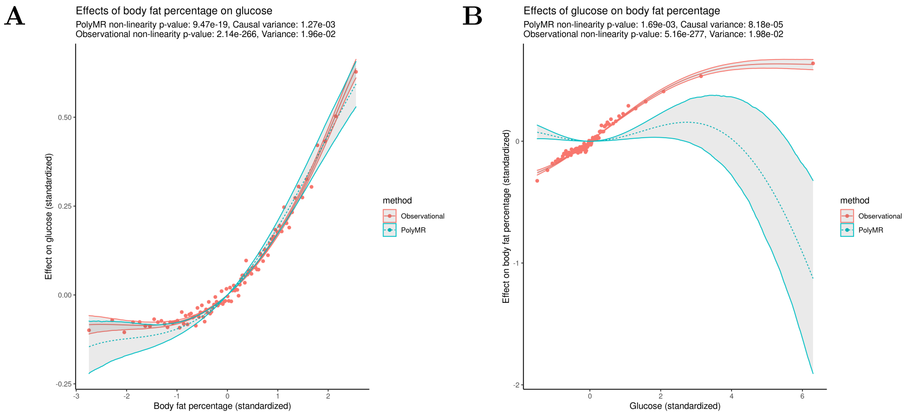

Supplementary figure 7: The observed (red, continuous) and causal (blue, dashed) association between body fat percentage and glucose (A) and the reverse (B). The observed association (red, continuous) was estimated using a similar polynomial approximation for the purpose of comparison, the points showing the mean outcome level at each percentile of the exposure. The hulls surrounding both functions show the 95% confidence range across the distribution.

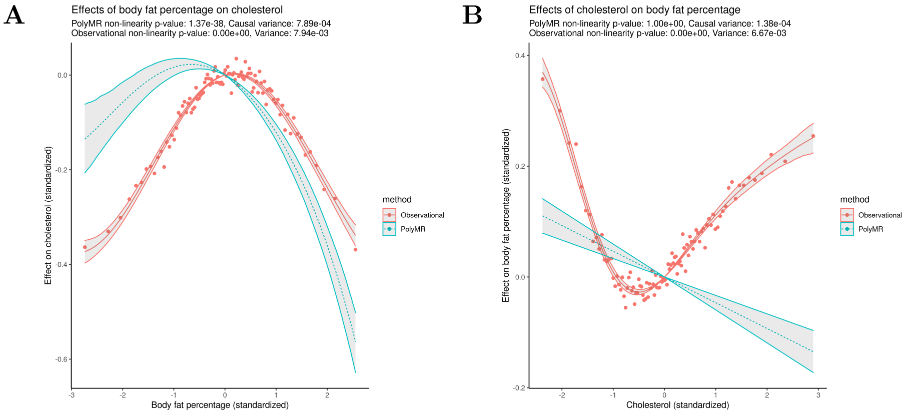

Supplementary figure 8: The observed (red, continuous) and causal (blue, dashed) association between body fat percentage and total cholesterol (A) and the reverse (B). The observed association (red, continuous) was estimated using a similar polynomial approximation for the purpose of comparison, the points showing the mean outcome level at each percentile of the exposure. The hulls surrounding both functions show the 95% confidence range across the distribution.

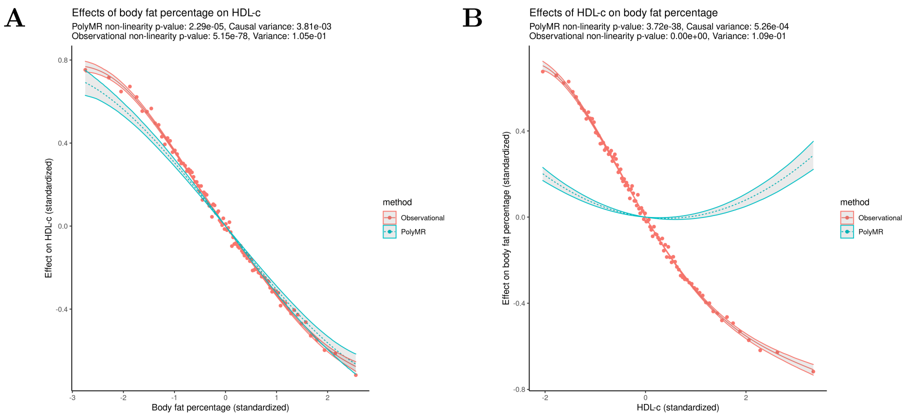

Supplementary figure 9: The observed (red, continuous) and causal (blue, dashed) association between body fat percentage and HDL cholesterol (A) and the reverse (B). The observed association (red, continuous) was estimated using a similar polynomial approximation for the purpose of comparison, the points showing the mean outcome level at each percentile of the exposure. The hulls surrounding both functions show the 95% confidence range across the distribution.

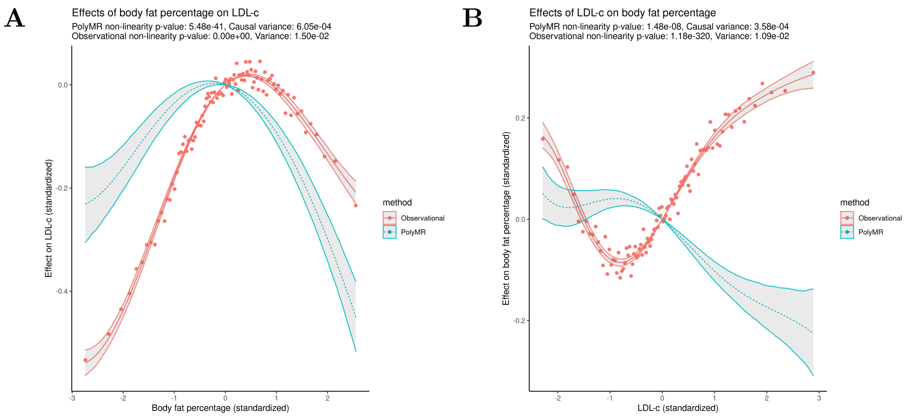

Supplementary figure 10: The observed (red, continuous) and causal (blue, dashed) association between body fat percentage and LDL cholesterol (A) and the reverse (B). The observed association (red, continuous) was estimated using a similar polynomial approximation for the purpose of comparison, the points showing the mean outcome level at each percentile of the exposure. The hulls surrounding both functions show the 95% confidence range across the distribution.

2.2 Waist-to-hip ratio

A B

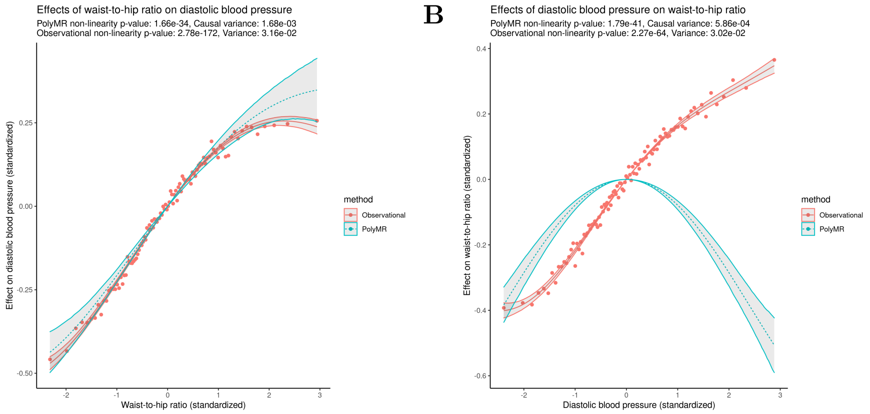

Supplementary figure 11: The observed (red, continuous) and causal (blue, dashed) association between waist-to-hip ratio and diastolic blood pressure (A) and the reverse (B). The observed association (red, continuous) was estimated using a similar polynomial approximation for the purpose of comparison, the points showing the mean outcome level at each percentile of the exposure. The hulls surrounding both functions show the 95% confidence range across the distribution.

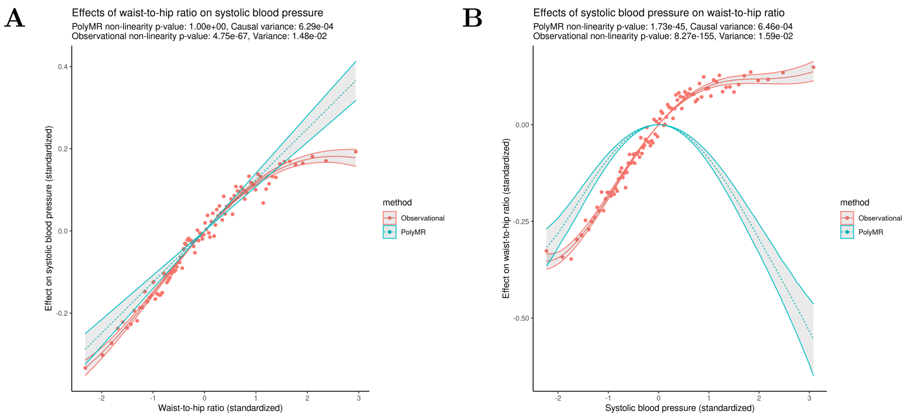

Supplementary figure 12: The observed (red, continuous) and causal (blue, dashed) association between waist-to-hip ratio and systolic blood pressure (A) and the reverse (B). The observed association (red, continuous) was estimated using a similar polynomial approximation for the purpose of comparison, the points showing the mean outcome level at each percentile of the exposure. The hulls surrounding both functions show the 95% confidence range across the distribution.

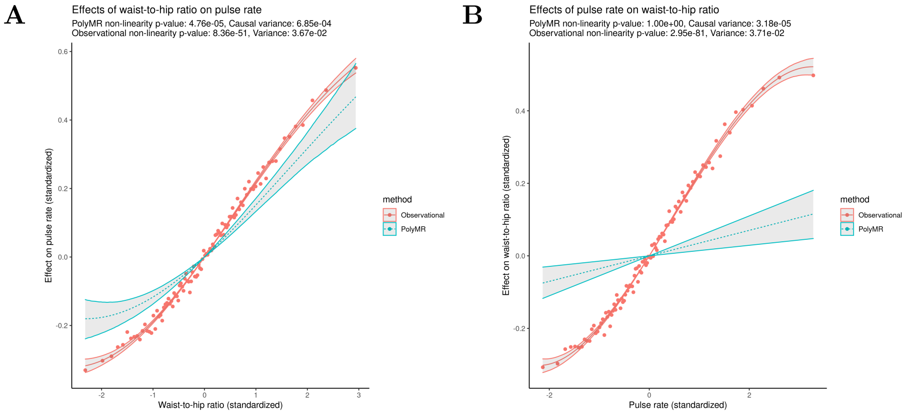

Supplementary figure 13: The observed (red, continuous) and causal (blue, dashed) association between waist-to-hip ratio and pulse rate (A) and the reverse (B). The observed association (red, continuous) was estimated using a similar polynomial approximation for the purpose of comparison, the points showing the mean outcome level at each percentile of the exposure. The hulls surrounding both functions show the 95% confidence range across the distribution.

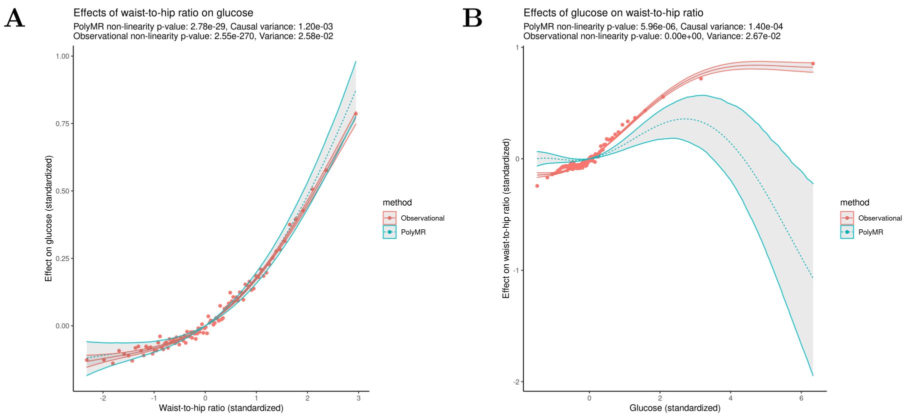

Supplementary figure 14: The observed (red, continuous) and causal (blue, dashed) association between waist-to-hip ratio and glucose (A) and the reverse (B). The observed association (red, continuous) was estimated using a similar polynomial approximation for the purpose of comparison, the points showing the mean outcome level at each percentile of the exposure. The hulls surrounding both functions show the 95% confidence range across the distribution.

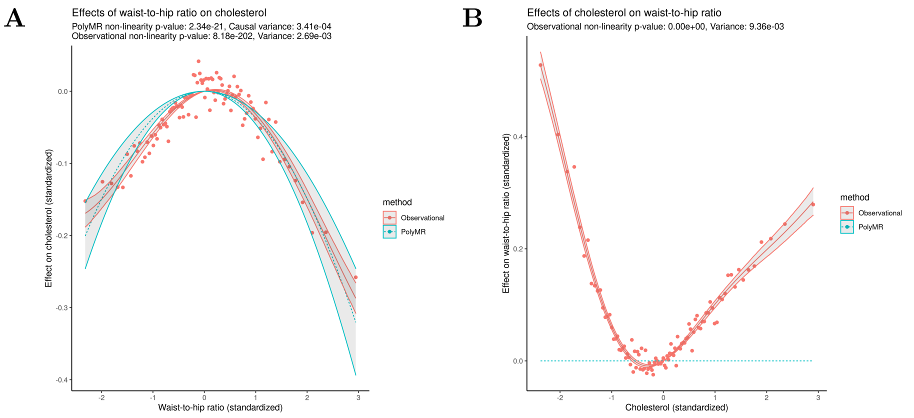

Supplementary figure 15: The observed (red, continuous) and causal (blue, dashed) association between waist-to-hip ratio and total cholesterol (A) and the reverse (B). The observed association (red, continuous) was estimated using a similar polynomial approximation for the purpose of comparison, the points showing the mean outcome level at each percentile of the exposure. The hulls surrounding both functions show the 95% confidence range across the distribution.

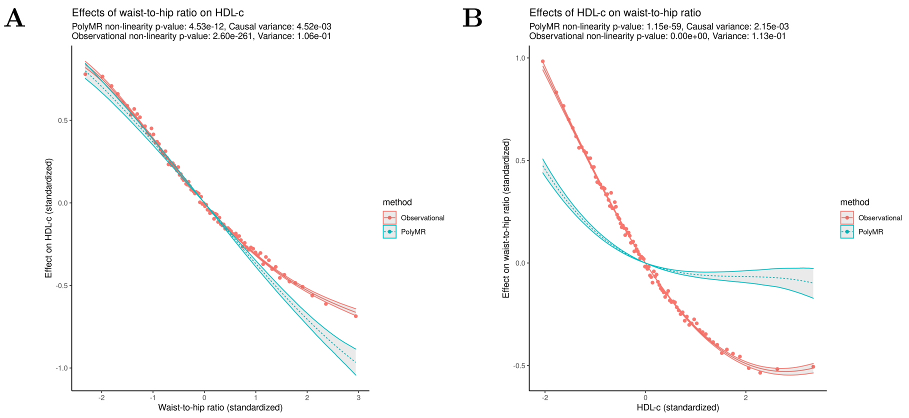

Supplementary figure 16: The observed (red, continuous) and causal (blue, dashed) association between waist-to-hip ratio and HDL cholesterol (A) and the reverse (B). The observed association (red, continuous) was estimated using a similar polynomial approximation for the purpose of comparison, the points showing the mean outcome level at each percentile of the exposure. The hulls surrounding both functions show the 95% confidence range across the distribution.

A

B

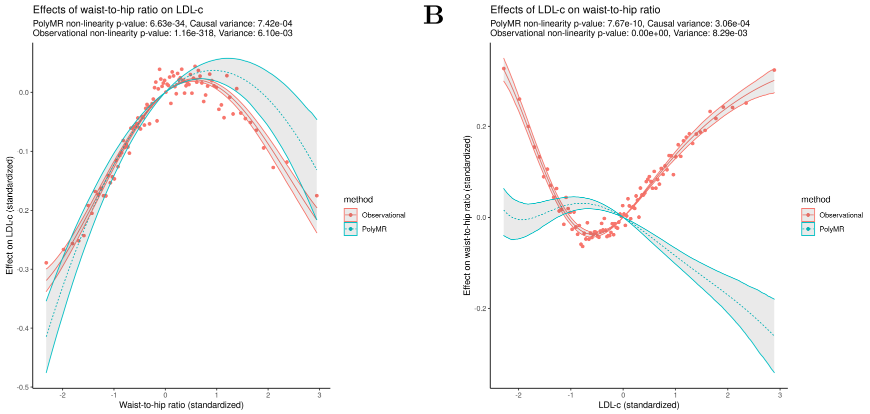

Supplementary figure 17: The observed (red, continuous) and causal (blue, dashed) association between waist-to-hip ratio and LDL cholesterol (A) and the reverse (B). The observed association (red, continuous) was estimated using a similar polynomial approximation for the purpose of comparison, the points showing the mean outcome level at each percentile of the exposure. The hulls surrounding both functions show the 95% confidence range across the distribution.

2.3 BMI

A **B**

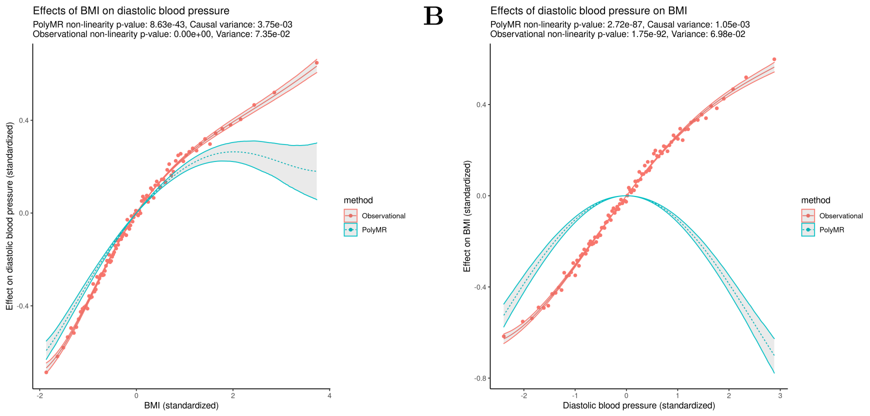

Supplementary figure 18: The observed (red, continuous) and causal (blue, dashed) association between BMI and diastolic blood pressure (A) and the reverse (B). The observed association (red, continuous) was estimated using a similar polynomial approximation for the purpose of comparison, the points showing the mean outcome level at each percentile of the exposure. The hulls surrounding both functions show the 95% confidence range across the distribution.

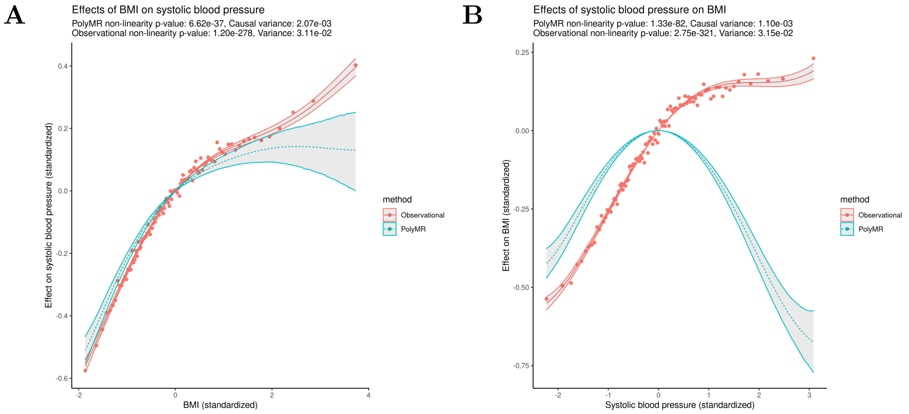

Supplementary figure 19: The observed (red, continuous) and causal (blue, dashed) association between BMI and systolic blood pressure (A) and the reverse (B). The observed association (red, continuous) was estimated using a similar polynomial approximation for the purpose of comparison, the points showing the mean outcome level at each percentile of the exposure. The hulls surrounding both functions show the 95% confidence range across the distribution.

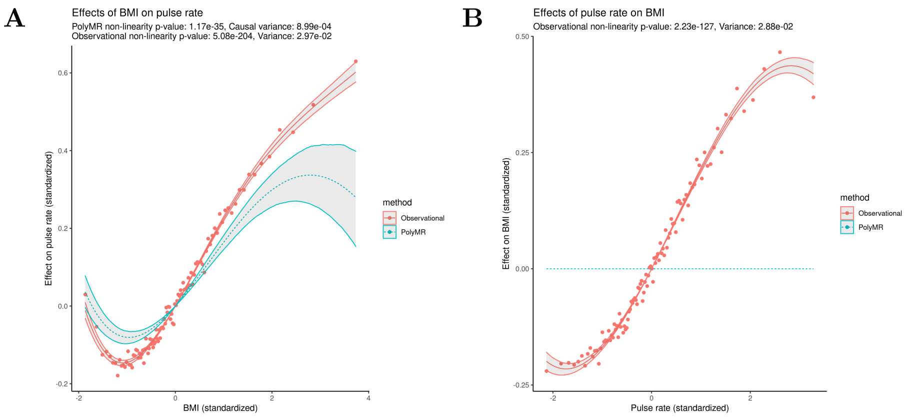

Supplementary figure 20: The observed (red, continuous) and causal (blue, dashed) association between BMI and pulse rate (A) and the reverse (B). The observed association (red, continuous) was estimated using a similar polynomial approximation for the purpose of comparison, the points showing the mean outcome level at each percentile of the exposure. The hulls surrounding both functions show the 95% confidence range across the distribution.

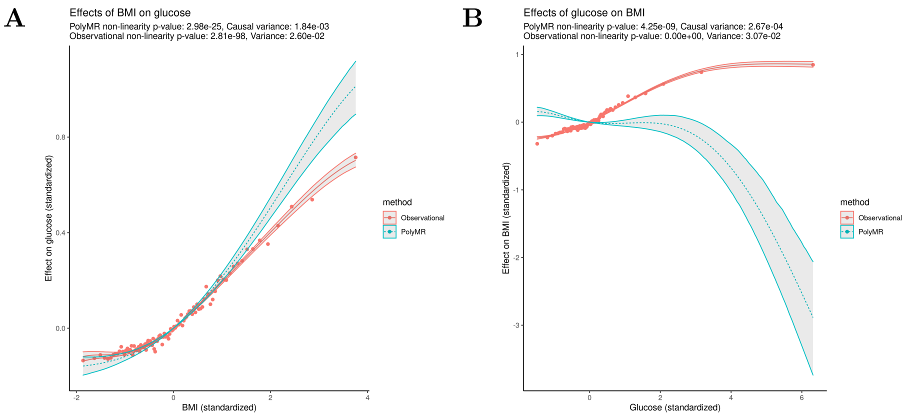

Supplementary figure 21: The observed (red, continuous) and causal (blue, dashed) association between BMI and glucose (A) and the reverse (B). The observed association (red, continuous) was estimated using a similar polynomial approximation for the purpose of comparison, the points showing the mean outcome level at each percentile of the exposure. The hulls surrounding both functions show the 95% confidence range across the distribution.

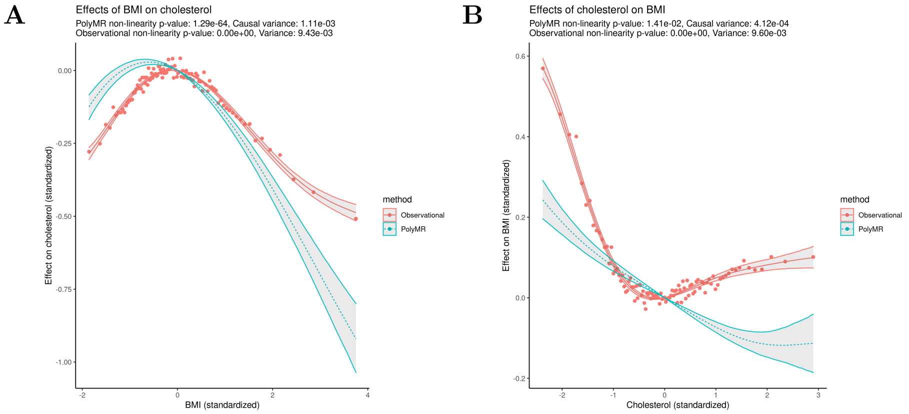

Supplementary figure 22: The observed (red, continuous) and causal (blue, dashed) association between BMI and total cholesterol (A) and the reverse (B). The observed association (red, continuous) was estimated using a similar polynomial approximation for the purpose of comparison, the points showing the mean outcome level at each percentile of the exposure. The hulls surrounding both functions show the 95% confidence range across the distribution.

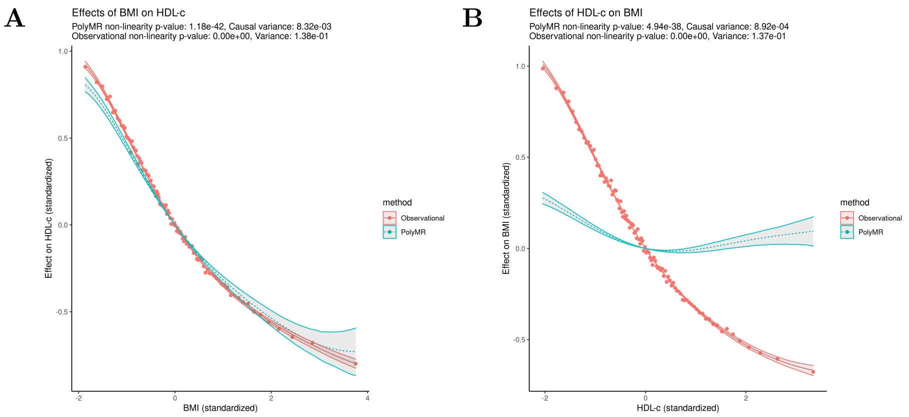

Supplementary figure 23: The observed (red, continuous) and causal (blue, dashed) association between BMI and HDL cholesterol (A) and the reverse (B). The observed association (red, continuous) was estimated using a similar polynomial approximation for the purpose of comparison, the points showing the mean outcome level at each percentile of the exposure. The hulls surrounding both functions show the 95% confidence range across the distribution.

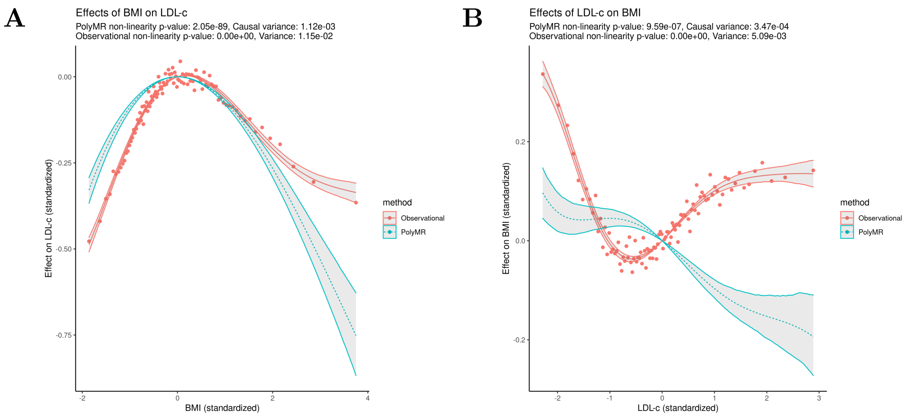

Supplementary figure 24: The observed (red, continuous) and causal (blue, dashed) association between BMI and LDL cholesterol (A) and the reverse (B). The observed association (red, continuous) was estimated using a similar polynomial approximation for the purpose of comparison, the points showing the mean outcome level at each percentile of the exposure. The hulls surrounding both functions show the 95% confidence range across the distribution.

2.4 Weight

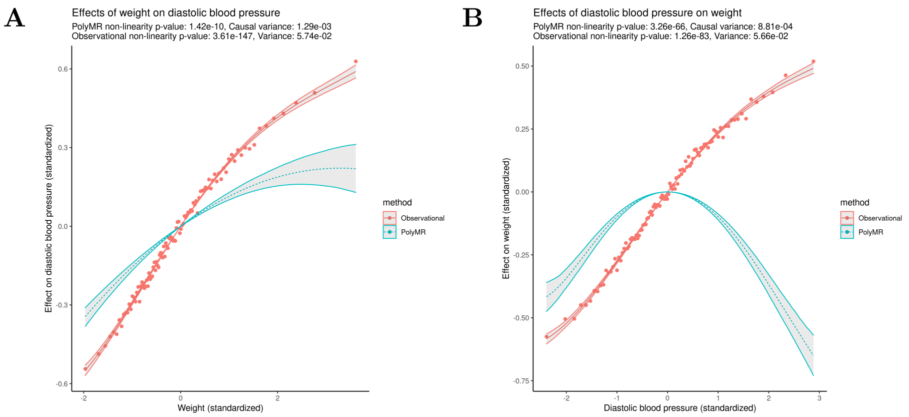

Supplementary figure 25: The observed (red, continuous) and causal (blue, dashed) association between weight and diastolic blood pressure (A) and the reverse (B). The observed association (red, continuous) was estimated using a similar polynomial approximation for the purpose of comparison, the points showing the mean outcome level at each percentile of the exposure. The hulls surrounding both functions show the 95% confidence range across the distribution.

A **B**

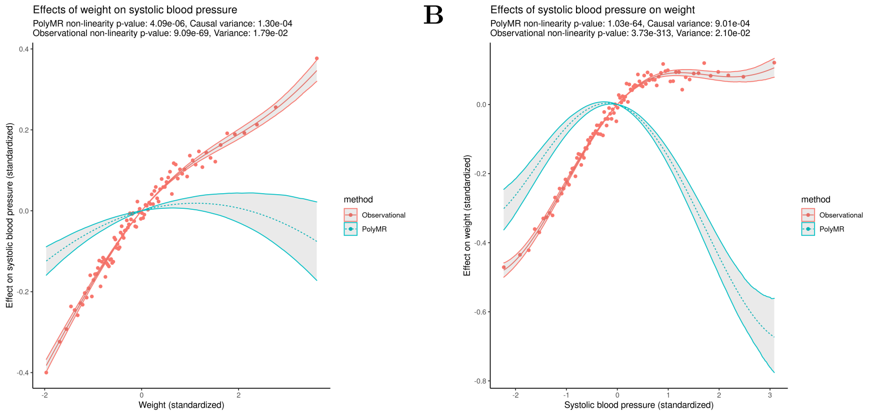

Supplementary figure 26: The observed (red, continuous) and causal (blue, dashed) association between weight and systolic blood pressure (A) and the reverse (B). The observed association (red, continuous) was estimated using a similar polynomial approximation for the purpose of comparison, the points showing the mean outcome level at each percentile of the exposure. The hulls surrounding both functions show the 95% confidence range across the distribution.

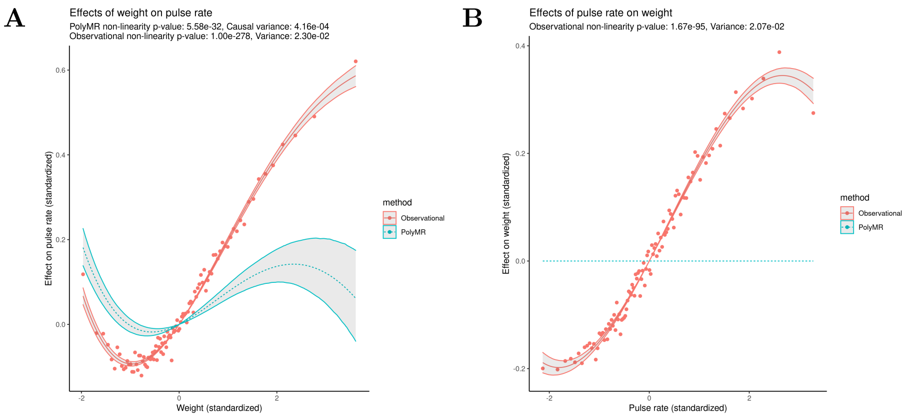

Supplementary figure 27: The observed (red, continuous) and causal (blue, dashed) association between weight and pulse rate (A) and the reverse (B). The observed association (red, continuous) was estimated using a similar polynomial approximation for the purpose of comparison, the points showing the mean outcome level at each percentile of the exposure. The hulls surrounding both functions show the 95% confidence range across the distribution.

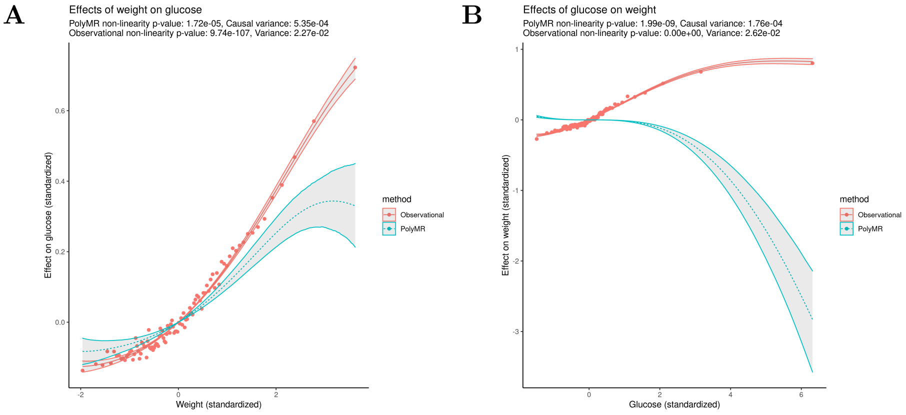

Supplementary figure 28: The observed (red, continuous) and causal (blue, dashed) association between weight and glucose (A) and the reverse (B). The observed association (red, continuous) was estimated using a similar polynomial approximation for the purpose of comparison, the points showing the mean outcome level at each percentile of the exposure. The hulls surrounding both functions show the 95% confidence range across the distribution.

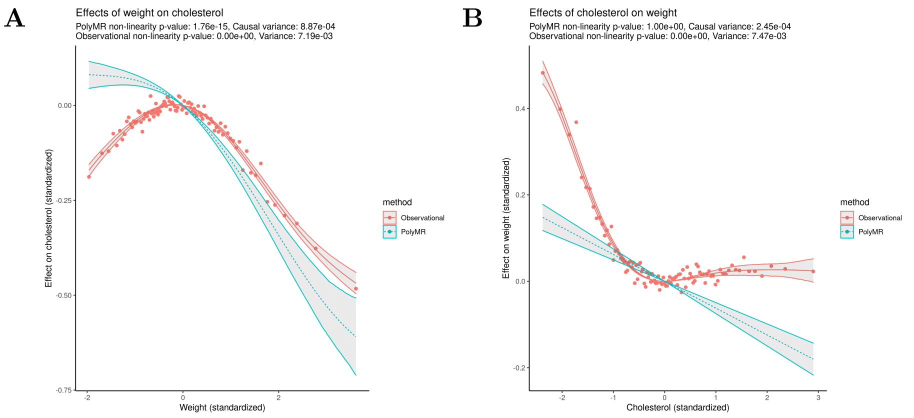

Supplementary figure 29: The observed (red, continuous) and causal (blue, dashed) association between weight and total cholesterol (A) and the reverse (B). The observed association (red, continuous) was estimated using a similar polynomial approximation for the purpose of comparison, the points showing the mean outcome level at each percentile of the exposure. The hulls surrounding both functions show the 95% confidence range across the distribution.

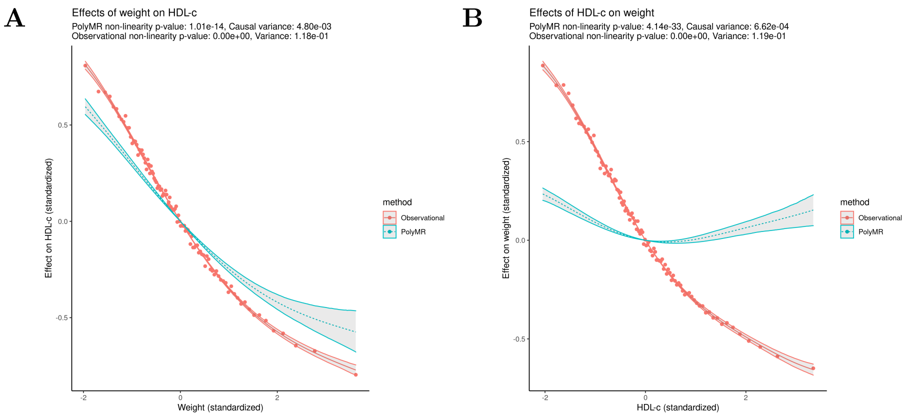

Supplementary figure 30: The observed (red, continuous) and causal (blue, dashed) association between weight and HDL cholesterol (A) and the reverse (B). The observed association (red, continuous) was estimated using a similar polynomial approximation for the purpose of comparison, the points showing the mean outcome level at each percentile of the exposure. The hulls surrounding both functions show the 95% confidence range across the distribution.

A

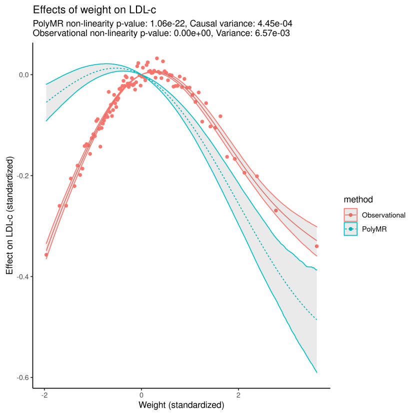

B

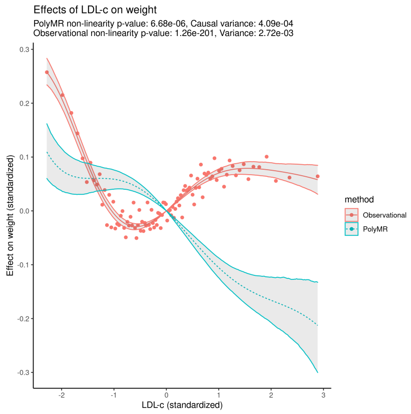

Supplementary figure 31: The observed (red, continuous) and causal (blue, dashed) association between weight and LDL cholesterol (A) and the reverse (B). The observed association (red, continuous) was estimated using a similar polynomial approximation for the purpose of comparison, the points showing the mean outcome level at each percentile of the exposure. The hulls surrounding both functions show the 95% confidence range across the distribution.

2.5 Education and life expectancy

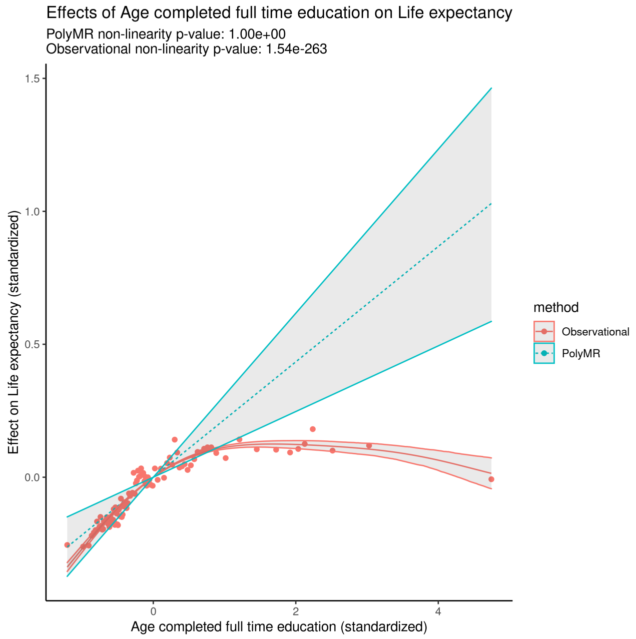

Supplementary figure 32: The observed (red, continuous) and causal (blue, dashed) association between age completed full-time education and life expectancy. The observed association (red, continuous) was estimated using a similar polynomial approximation for the purpose of comparison, the points showing the mean outcome level at each percentile of the exposure. The hulls surrounding both functions show the 95% confidence range across the distribution.

3 PolyMR-L1

3.1 BMI

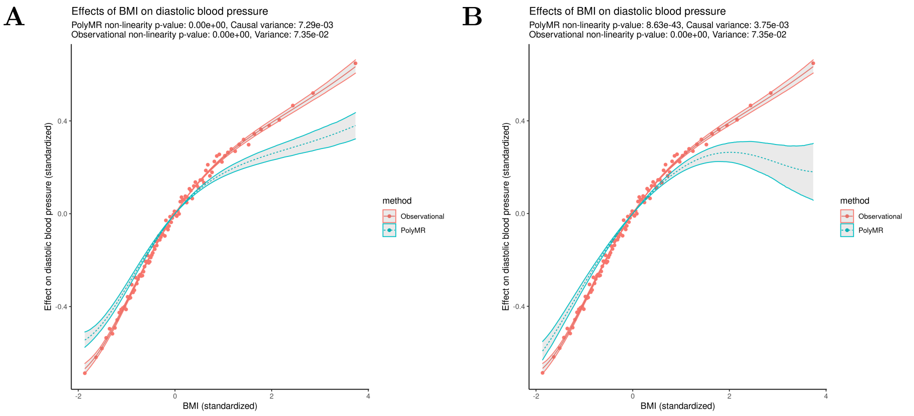

Supplementary figure 33: The observed (red, continuous) and causal (blue, dashed) association between BMI and diastolic blood pressure, using PolyMR-L1 (accounting for linear confounding alone, A) and PolyMR (B). The observed association (red, continuous) was estimated using a similar polynomial approximation for the purpose of comparison, the points showing the mean outcome level at each percentile of the exposure. The hulls surrounding both functions show the 95% confidence range across the distribution. Plot B is reproduced here for convenience but is identical to that included above.

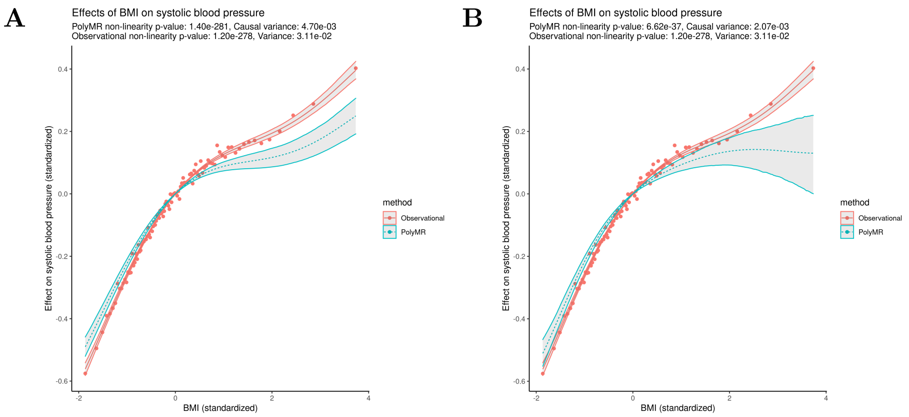

Supplementary figure 34: The observed (red, continuous) and causal (blue, dashed) association between BMI and systolic blood pressure, using PolyMR-L1 (accounting for linear confounding alone, A) and PolyMR (B). The observed association (red, continuous) was estimated using a similar polynomial approximation for the purpose of comparison, the points showing the mean outcome level at each percentile of the exposure. The hulls surrounding both functions show the 95% confidence range across the distribution. Plot B is reproduced here for convenience but is identical to that included above.

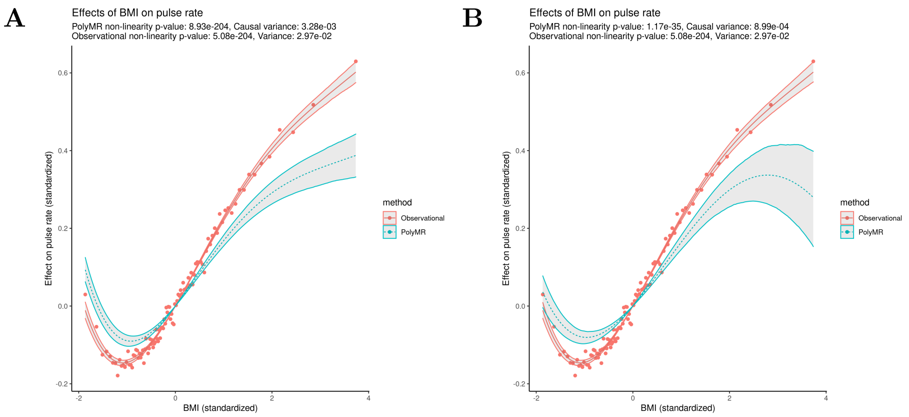

Supplementary figure 35: The observed (red, continuous) and causal (blue, dashed) association between BMI and pulse rate, using PolyMR-L1 (accounting for linear confounding alone, A) and PolyMR (B). The observed association (red, continuous) was estimated using a similar polynomial approximation for the purpose of comparison, the points showing the mean outcome level at each percentile of the exposure. The hulls surrounding both functions show the 95% confidence range across the distribution. Plot B is reproduced here for convenience but is identical to that included above.

A B

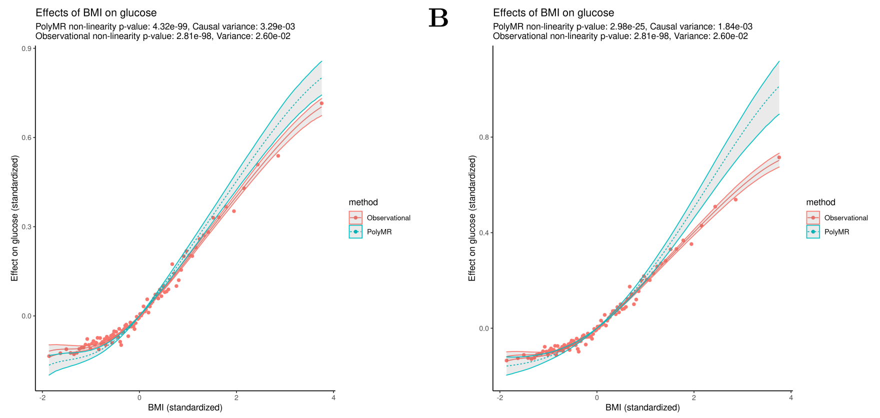

Supplementary figure 36: The observed (red, continuous) and causal (blue, dashed) association between BMI and glucose, using PolyMR-L1 (accounting for linear confounding alone, A) and PolyMR (B). The observed association (red, continuous) was estimated using a similar polynomial approximation for the purpose of comparison, the points showing the mean outcome level at each percentile of the exposure. The hulls surrounding both functions show the 95% confidence range across the distribution. Plot B is reproduced here for convenience but is identical to that included above.

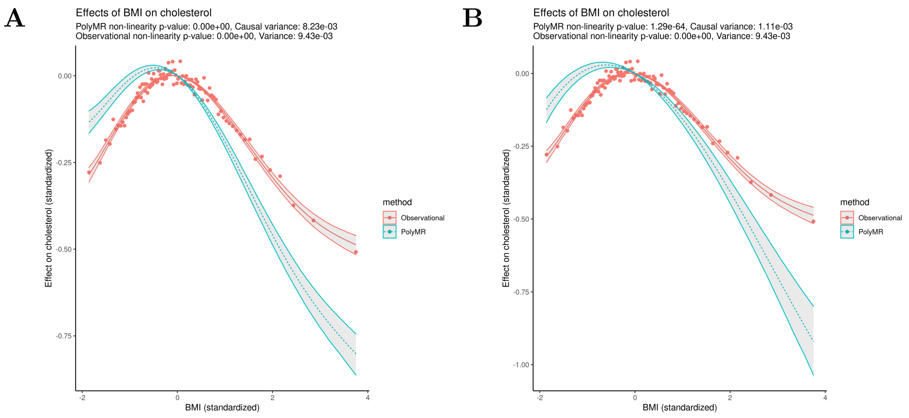

Supplementary figure 37: The observed (red, continuous) and causal (blue, dashed) association between BMI and total cholesterol, using PolyMR-L1 (accounting for linear confounding alone, A) and PolyMR (B). The observed association (red, continuous) was estimated using a similar polynomial approximation for the purpose of comparison, the points showing the mean outcome level at each percentile of the exposure. The hulls surrounding both functions show the 95% confidence range across the distribution. Plot B is reproduced here for convenience but is identical to that included above.

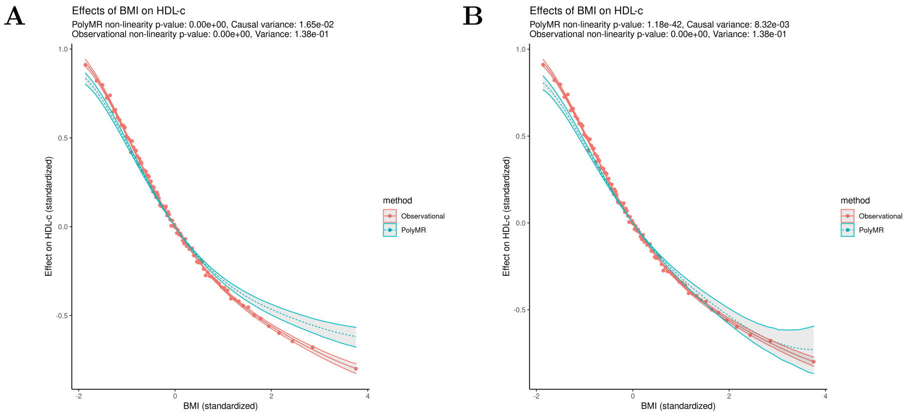

Supplementary figure 38: The observed (red, continuous) and causal (blue, dashed) association between BMI and HDL cholesterol, using PolyMR-L1 (accounting for linear confounding alone, A) and PolyMR (B). The observed association (red, continuous) was estimated using a similar polynomial approximation for the purpose of comparison, the points showing the mean outcome level at each percentile of the exposure. The hulls surrounding both functions show the 95% confidence range across the distribution. Plot B is reproduced here for convenience but is identical to that included above.

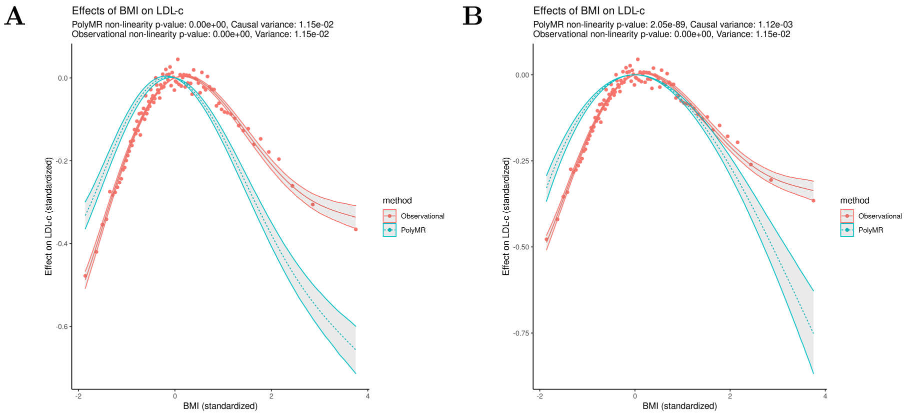

Supplementary figure 39: The observed (red, continuous) and causal (blue, dashed) association between BMI and LDL cholesterol, using PolyMR-L1 (accounting for linear confounding alone, A) and PolyMR (B). The observed association (red, continuous) was estimated using a similar polynomial approximation for the purpose of comparison, the points showing the mean outcome level at each percentile of the exposure. The hulls surrounding both functions show the 95% confidence range across the distribution. Plot B is reproduced here for convenience but is identical to that included above.
